# CGRP Receptor Family and Accessory Protein Localization: Implications for Predicted Function

**DOI:** 10.1100/tsw.2001.431

**Published:** 2001-06-05

**Authors:** K.R. Oliver

**Affiliations:** Merck Research Laboratories, Neuroscience Research Centre, Harlow, Essex CM20 2QR, UK

## Abstract

Calcitonin gene-related peptide (CGRP), adrenomedullin, amylin, and calcitonin are functionally related neuropeptides. Certain of these peptides mediate their action through receptors which have common components, such as the receptor activity modifying proteins (RAMPs) and CGRP-receptor component protein, as well as possibly through other distinct receptors. Specifically, the molecular pharmacology of CGRP and adrenomedullin is determined by coexpression of one of three receptor activity-modifying proteins (RAMPs) with calcitonin receptor-like receptor (CRLR). Additionally, through formation of another hetero-oligomer, RAMPs also govern the pharmacology of the calcitonin receptor, which in association with RAMP1 or RAMP3, binds amylin with high affinity. We have used multiple approaches to discern the regional and cellular expression of these various receptor components and binding sites for the above neuropeptides in multiple species and in different tissues. Techniques applied include in situ hybridization, immunohistochemistry and radioligand autoradiography. These data allow further understanding of both the complexity of receptor-receptor component and receptor-ligand interactions in vivo. Interestingly, these localization data suggest that RAMPs may interact with receptors additional to those already identified for the CGRP family and may be involved in binding innate neuropeptides or other neurotransmitters which are not members of the calcitonin gene-related peptide fam

